# Direct aldosterone stimulation of skeletal muscle fibroblasts changes gene expression and differentially affects fibroblast functions from normal and diseased muscles

**DOI:** 10.3389/fphys.2026.1760238

**Published:** 2026-04-20

**Authors:** Chetan K. Gomatam, Swathy Krishna, Jeovanna Lowe, Esther Silver, Christoph Lepper, Jill A. Rafael-Fortney

**Affiliations:** Department of Physiology and Cell Biology, College of Medicine, The Ohio State University, Columbus, OH, United States

**Keywords:** Duchenne muscular dystrophy, fibroblasts, fibrosis, *mdx*, mineralocorticoid receptor

## Abstract

**Introduction:**

Fibroblasts are critical for stabilizing skeletal muscle and facilitating wound healing after injury but become overactivated and lead to fibrotic replacement of muscle tissue in chronic degenerative diseases such as Duchenne muscular dystrophy (DMD). We have previously shown that the mineralocorticoid receptor (MR) is present in skeletal muscle and MR antagonist drugs reduce fibrosis and chronic inflammation in dystrophic mouse models. Indirect MR signaling from other cell types in the muscle microenvironment affects fibroblast gene expression and function. However, the direct effects of MR activation of skeletal muscle fibroblasts are unknown.

**Methods:**

To determine whether direct stimulation with the endogenous MR agonist aldosterone changes gene expression in skeletal muscle fibroblasts, we performed RNA sequencing comparing fibroblasts isolated from neonatal wild-type skeletal muscles treated with aldosterone or vehicle. To further investigate the effects of aldosterone treatment of fibroblasts in skeletal muscle health and disease, we then performed *in vitro* proliferation and migration assays on fibroblasts isolated from neonatal and adult wild-type and dystrophic muscles.

**Results:**

Treatment with aldosterone leads to differential expression of 492 genes in fibroblasts isolated from neonatal wild-type mouse muscles. Protein levels of differentially expressed genes Fkbp5, p57 and c-Fos were also increased by direct aldosterone stimulation of fibroblasts from both wild-type and dystrophic muscles. Surprisingly, cultured fibroblasts from both neonatal wild-type and dystrophic muscles retain a higher proliferation rate compared to adult muscle fibroblasts. Direct aldosterone treatment represses proliferation and slows scratch-wound closure kinetics only in fibroblasts isolated from adult dystrophic skeletal muscle.

**Discussion:**

This study shows that aldosterone treatment of skeletal muscle fibroblasts alters gene expression. However, fibroblasts from adult dystrophic muscle appear most sensitive to gene expression changes after short-term aldosterone treatment. These data suggest that MR signaling in the skeletal muscle microenvironment may differentially affect fibroblasts in wound healing and in chronic fibrotic diseases such as muscular dystrophies.

## Introduction

Fibroblasts are critical for effective wound healing in skeletal muscle. As the main extracellular matrix-producing cell type in the muscle microenvironment, they are responsible for scaffolding and stabilizing muscle after an acute injury to allow for satellite cell migration and muscle fiber regeneration (Klingler et al., 2012; Bentzinger et al., 2013; Tidball, 2017). Transient upregulation of extracellular matrix (ECM) components during regeneration after an acute injury does not typically result in permanent or prominent scar tissue.

In chronic muscle diseases such as Duchenne muscular dystrophy (DMD), however, muscle fiber membranes are prone to contraction-induced damage due to the lack of the dystrophin protein. Dystrophin normally recruits the dystrophin-glycoprotein complex to connect the actin cytoskeleton to laminin in the basement membrane and helps distribute contractile force (Ahn and Kunkel, 1993). Repeated cycles of muscle fiber degeneration and regeneration in the absence of dystrophin lead to overactivation of immune cells and fibroblasts that respond to injuries, causing chronic inflammation and fibrosis that eventually replaces healthy muscle tissue (Tidball et al., 2018; Tidball, 2017).

We have previously demonstrated that the mineralocorticoid receptor (MR) is a potential therapeutic target for dystrophic skeletal muscle (Rafael-Fortney et al., 2011; Chadwick et al., 2015; Lowe et al., 2016, 2020). While MR is canonically known as a regulator of blood pressure and electrolyte homeostasis, we have shown that it is present in skeletal muscle and that MR antagonism leads to beneficial effects such as decreased inflammatory signaling and fibrosis and increased muscle force in dystrophic mouse models (Delyani et al., 2001; Tamargo et al., 2014; Rafael-Fortney et al., 2011; Chadwick et al., 2015; Lowe et al., 2016, 2020). The MR antagonist (MRA) spironolactone also increases membrane stability through a mechanism independent of myofiber MR (Chadwick et al., 2017a; Hauck et al., 2019b). MRAs have also been shown to improve cardiomyopathy in DMD patients and have become a recommended standard-of-care (Raman et al., 2017, 2015, 2019; Buddhe et al., 2018; Haddad et al., 2023).

Infiltrating immune cells, transiently present in wild-type muscles after an acute injury and chronically present in dystrophic muscles, express Cyp11b2 (aldosterone synthase) and produce high local levels of the canonical MR agonist aldosterone within damaged muscles (Chadwick et al., 2016; Hauck et al., 2019a). Aldosterone binding translocates MR to the nucleus where it acts as a transcription factor for many profibrotic and pro-inflammatory genes (Viengchareun et al., 2007). Dystrophic muscles become increasingly aldosterone selective by producing 11β-HSD2 that inactivates the high levels of circulating cortisol/corticosterone that can also activate MR (Chadwick et al., 2016).

Using cell-specific knockouts of MR in the dystrophin-deficient *mdx* mouse model of DMD, we have also shown that MR signaling in myofibers and myeloid immune cells differentially affect fibrosis (Hauck et al., 2019b; Howard et al., 2022b; Gomatam et al., 2024). We have recently demonstrated that secreted factors from MR stimulated differentiated cultured muscle fibers (myotubes) alter gene expression of muscle fibroblasts (Krishna et al., 2025). These data show that MR signaling in other cell types in the skeletal muscle microenvironment indirectly affect fibroblast gene expression and function. However, the direct effects of MR signaling on muscle fibroblast gene expression and function have not yet been explored. It is known that treatment of cardiac and renal fibroblasts with aldosterone increases fibroblast gene expression, migration, proliferation, and extracellular matrix production, and that MRA treatment inhibits these direct effects (Somanna et al., 2015; Stockand and Meszaros, 2003; Huang et al., 2012).

In the current study, we tested whether direct treatment of fibroblasts with aldosterone altered gene expression in neonatal mouse skeletal muscle fibroblasts. We then tested the effects of aldosterone on fibroblast proliferation and migration in neonatal and adult wild-type and *mdx* fibroblasts to determine whether direct MR stimulation differentially affects muscle fibroblasts in health and disease. Our data demonstrate that MR signaling directly impacts skeletal muscle fibroblast transcriptome and function. Understanding the direct role of MR signaling in fibroblasts and the subsequent impact on fibroblast functions is crucial for developing therapies for skeletal muscle diseases that do not compromise normal fibroblast-mediated wound healing. Of note, fibroblasts in our study were isolated based on their ability to adhere to a substrate and have been shown to be equivalent to the cell type also known as fibroadipogenic progenitors (FAPs) from skeletal muscle (Contreras et al., 2019). Fibroblast populations isolated from *mdx* and wild-type skeletal muscle based on substrate adherence have been shown to be 80-85% positive for the fibroblast marker PDGFRα (Contreras et al., 2019). The passage 2 cells used in the current study further enrich for fibroblasts. We will subsequently refer to these adherence-enriched fibroblasts/FAPs as “fibroblasts” for consistency.

## Materials and methods

### Animals

All mouse protocols were approved by the Institutional Animal Care and Use Committee of the Ohio State University, comply with all laws of the United States of America, and conform to the National Institutes of Health Guide for the Care and Use of Laboratory Animals. C57BL/10 (C57) wild-type and *mdx* mice were bred in house as separate mouse lines.

### Fibroblast isolation

Fibroblasts were isolated from the hindlimb muscles of three-day-old C57 wild-type neonatal mice. The hindlimbs were dissected, skinned, rinsed in ice-cold 1X Dulbecco’s Phosphate Buffered Saline without calcium and magnesium (DPBS) (Life Technologies/Gibco, Waltham, MA, USA, 14190-144), and digested with 5 ml of digestion buffer consisting of 0.1% Type II collagenase (Sigma-Aldrich, St. Louis, MO, USA, C6885-500MG), 0.1% BSA (Fisher Scientific, Pittsburgh, PA, USA, BP1605-100), and 0.1% DNase I (RQ1; Promega, Madison, WI, USA, M610A) diluted in DPBS (Life Technologies/Gibco, Waltham, MA, USA, 14190-144) for 45 minutes in a 37 °C water bath, with trituration using a fetal bovine serum (FBS)-coated pipette every 15 minutes. When only bone remained, the digestion solution was neutralized with 3 ml of fibroblast growth media [DMEM (Thermo Fisher Scientific, Waltham, MA, USA, 11995-065), 10% FBS (Atlanta Biologicals [now R&D Systems], Minneapolis, MN, USA, S11150), and 1% penicillin-streptomycin (Life Technologies/Gibco, Waltham, MA, USA, 15140-122)], and the muscle suspensions were filtered through 70-micron filters (Fisher Scientific, Pittsburgh, PA, USA, 22-363-548). The filters were rinsed with an additional 2 ml of fibroblast growth media before the suspensions were filtered through 40-micron filters (Fisher Scientific, Pittsburgh, PA, USA, 22-363-547) and centrifuged at 1000 RPM at RT for 10 minutes. Cell pellets were resuspended in 1 ml of fibroblast growth media, then adherence purified via plating on a 10 cm tissue culture-treated plate (CytoOne Dishes, USA Scientific, Ocala, FL, USA, CC7682-3394) for 2 hours to enrich for fibroblasts. Cells were lifted with 2 ml of 1x Trypsin/EDTA (Sigma-Aldrich, St. Louis, MO, USA, 5428C-500ml) and neutralized with 3 ml of fibroblast growth media; then centrifuged at 1000 RPM for 10 minutes at room temperature. Afterwards, the adherence-purified fibroblasts were resuspended in 2 ml of fibroblast growth media and cell counted with trypan blue (Sigma-Aldrich, St. Louis, MO, USA, T8154-20ml) exclusion using a hemocytometer. Fibroblasts were plated on tissue culture-treated plates and allowed to replicate for several days before collecting and freezing down at a concentration of 277,500 cells/ml.

### Fibroblast treatment

Fibroblasts were thawed and cultured at 37° C and 5% CO2 in fibroblast growth media for 3 days in 10 cm tissue culture-treated plates. For 24 hours before treatment, the fibroblasts were cultured in fibroblast growth media containing FBS stripped by activated charcoal (Sigma-Aldrich, St. Louis, MO, USA, 05105-250G) to remove endogenous steroids. The fibroblasts were then pooled for each mouse and re-plated in new 10 cm tissue culture-treated plates for treatment in fibroblast growth media with charcoal-stripped FBS. Four plates each with 300,000 fibroblasts were treated with either aldosterone (final concentration of 1 μM) (Sigma-Aldrich, St. Louis, MO, USA, A9477-5MG), spironolactone (final concentration of 1 μM) (Sigma-Aldrich, St. Louis, MO, USA, S3378-5G), or vehicle (DMSO, Sigma-Aldrich, St. Louis, MO, USA, D2650-100ml) for 48 hours. Though 1 µM is higher than the average physiological level of aldosterone in skeletal muscle, this dose has been used in previous studies in different cell types, including cardiomyocytes and cardiac fibroblasts, to elicit maximal gene expression and protein level changes of profibrotic and proinflammatory genes of interest with short-term treatment (Somanna et al., 2015; Mummidi et al., 2016). Additionally, local production of aldosterone in diseased cardiac muscle and in dystrophic skeletal muscle by Cyp11B2 from infiltrating myeloid cells can reach similar levels, so this dosage is close to pathophysiological levels of aldosterone (Maron et al., 2016; Mummidi et al., 2016; Chadwick et al., 2016). Spironolactone has been used at various concentrations to treat different cell types *in vitro*, ranging from less than 1 µM to 50 µM (Williams et al., 2006; Kemp et al., 2019; Gold et al., 2019). For the sake of consistency with aldosterone dosage, and since spironolactone served as a control in our experiments for the ability of charcoal-stripped serum to remove endogenous steroids, we used 1 µM as the concentration of spironolactone. 10 mM stock solutions of aldosterone and spironolactone in DMSO were made by adding 3.60 mg (0.01 mmol) of aldosterone (MW: 360.44 g/mol) and 4.20 mg (0.01 mmol) of spironolactone (416.57 g/mol) to 1 ml of 100% DMSO, respectively. One μl of either 10 mM aldosterone in DMSO, 10 mM spironolactone in DMSO, or 100% DMSO as the vehicle was added to 10 ml of media in each 10 cm plate for final aldosterone and spironolactone concentrations of 1 μM and final DMSO concentrations of 0.01% v/v.

### RNA isolation and bulk RNA sequencing

After treatment, the fibroblasts were washed with warm DPBS, lysed directly in the plates with 1 ml TRIzol reagent (Invitrogen, Waltham, MA, USA, 15596026) and detached from the plates using a cell scraper (CytoOne Dishes, USA Scientific, Ocala, FL, USA, CC7600-0320). RNA was isolated and purified from each sample (n=4 per group) using the Direct-zol RNA microprep kit (Zymo Research, Irvine, CA, USA, R2060) according to manufacturer instructions. RNA purity and integrity were verified using a NanoDrop 2000c spectrophotometer (Thermo Fisher Scientific, Waltham, MA, USA) and an Agilent 5400 Bioanalyzer (Agilent Technologies, Santa Clara, CA, USA). All samples had RNA Integrity Numbers from 8.2-9.6. RNA samples from each of the 4 aldosterone-, 4 vehicle- and 4 spironolactone-treated fibroblast samples were prepared at a concentration of 50 ng/μl and shipped to Novogene Co., Ltd. (Davis, CA), for additional quality control, library preparation, and sequencing. Briefly, libraries were prepared using polyA enrichment, checked with Qubit and real-time PCR for quantification and on an Agilent bioanalyzer for size distribution detection. Sequencing was then performed using the Illumina NovaSeq X Plus platform. Alignment was performed using HISAT2, and the reference genome used was mm9 (NCBI RefSeq assembly: GCF_000001635.18). Differential expression analysis was performed using DESeq2 in Novogene’s NovoMagic software with significance established at |log_2_ fold change| ≥ 1 and p_adj_ ≤ 0.05 using Benjamini-Hochberg FDR correction. Novomagic was also used to perform heatmap and dot plot generation. To further understand the overall contribution of various genes to fibroblast function, we performed Gene Set Enrichment Analysis (GSEA) v4.4.0 (Subramanian et al., 2005; Mootha et al., 2003). The gene counts obtained from NovoMagic were used for the analyses with default parameters. Terms with FDR (q-value) < 0.1 and normalized enrichment score (NES) > 1.6 were designated as significantly enriched. These significantly enriched terms were further consolidated using an R package, GOSemSim (Yu et al., 2010; Yu, 2020), that performed a semantic similarity analysis. Hierarchical clustering of the terms was performed (**Supplementary Figure 1**), and a cut-off of h = 0.7 was used for consolidating the terms. All sequencing data have been deposited to the NIH NCBI Gene Expression Omnibus (accession no. GSE297237).

### Western blots

Western blots were performed to test protein levels for representative genes identified as differentially expressed by RNA sequencing. Protein was isolated from passage 2 cultured fibroblasts isolated from hindlimb muscle of neonatal C57 wild-type (n = 3; 2 M, 1 F) and *mdx* (n = 3; 2 M, 1 F) mice and treated with either aldosterone (1 µM) or vehicle, as above or with TGF-β1 (10 ng/ml) (Bon-Opus Biosciences, Milburn, NJ, USA, CA59) for 48 hours. Protein was also isolated from passage 2 cultured fibroblasts isolated from quadriceps muscle of adult C57 wild-type (n = 3; 2 M, 1 F) and *mdx* (n = 3; 2 M, 1 F) mice and treated with either aldosterone (1 µM) or vehicle for 48 hours as above. Protein isolated from flash-frozen quadriceps from adult (8-week-old) C57 and *mdx* mice was used to test antibodies and to test association of differential expression with disease. Protein was resuspended in either Newcastle buffer (4M Urea, 3.8% SDS, 75 mM Tris pH 6.8, 20% glycerol) (for Fkbp5) or cellular extract (CE) buffer (10 mM HEPES pH 7.6, 60 mM potassium chloride, 1 mM EDTA, 0.25% Tergitol-type NP-40 (for c-Fos and p57 Kip2) as previously described (Chadwick et al., 2015). 10 µg of neonatal and adult fibroblast protein and 100 µg of adult protein from quadriceps were run on 10% SDS-PAGE and transferred to nitrocellulose membranes. Ponceau S reagent (Sigma-Aldrich, St. Louis, MO, USA, P7170-1L) was used to stain total protein before antibody probing and was used as a normalization control. Aldosterone treatment affects expression of housekeeping proteins such as GAPDH, so these proteins were not viable as controls. Membranes were incubated in rabbit primary antibodies against FK506-binding protein 51 (Fkbp5, 1:1000) (Invitrogen, Waltham, MA, USA, 702260), p57 Kip2 (p57, 1:500) (Invitrogen, Waltham, MA, USA, MA5-56534), and c-Fos (0.2 µg/ml) (Developmental Studies Hybridoma Bank, University of Iowa, Iowa City, IA, USA, CPTC-FOS-1-S deposited by Clinical Proteomics Technologies for Cancer). Horseradish peroxidase (HRP)-conjugated goat anti-rabbit (1:10,000) (Jackson ImmunoResearch Labs, West Grove, PA, 111-035-144) was used as secondary antibody for Fkbp5 and p57, and HRP-goat anti-mouse (1:10,000) (Jackson ImmunoResearch Labs, West Grove, PA, 115-035-146) was used as secondary antibody for c-Fos. Secondary antibodies were detected using an ECL2 kit (Pierce Biotechnology, Inc., Waltham, MA, USA, 80196) followed by exposure using the Chemidoc MP Imaging system (Bio-Rad Laboratories, Hercules, CA).

Quantification was performed using the ImageLab software (Bio-Rad Laboratories, Hercules, CA). Total protein staining was used as a normalization control for all proteins. Briefly, the Lane tool was used to quantify the total protein present in each lane in arbitrary units. The Band tool was used to quantify individual bands of the protein of interest. The values obtained for the proteins of interest were then divided by their corresponding total protein values to normalize the protein quantification.

### EdU proliferation assay

EdU proliferation assays were performed with fibroblasts isolated from quadriceps (adult) or hindlimb (neonatal) skeletal muscle from adult (9-week-old) C57 wild-type mice (n= 3; 1 M, 2 F), adult (9-week-old) *mdx* mice (n = 4; 2 M, 2 F), neonatal (4 days postnatal) C57 wild-type mice (n = 3), and neonatal (2 days postnatal) *mdx* mice (n = 3). Primary fibroblasts were cultured with fibroblast growth media in tissue culture plates as described above until passage 2. Twenty-four hours before treatment, the fibroblasts were incubated at 37 °C and 5% CO_2_ in fibroblast growth media containing 10% charcoal-stripped FBS to remove endogenous steroids. Fibroblasts were then plated with the charcoal-stripped media in 4-well glass chamber slides (Nunc Lab-Tek II chamber slides, Thermo Fisher Scientific, Waltham, MA, USA, 154526) at 10,000 cells/well for treatment. The fibroblasts were treated for 48 hours at 37 °C and 5% CO_2_ with 1 μM aldosterone, 1 μM spironolactone, or vehicle (DMSO) in 500 µl of growth media with charcoal-stripped serum as above. Treatment with 10 ng/ml TGF-β1 (Bon-Opus Biosciences, Milburn, NJ, USA, CA59) served as a positive control, and three wells were used as no-cell controls to ensure that there was no contamination in the media.

The following components were used from the Click-iT EdU Alexa Fluor 647 Imaging Kit (Invitrogen, Waltham, MA, USA, C10340): EdU (5-ethynyl-2’-deoxyuridine), Alexa Fluor 647 azide (sulfo-cyanine 5 azide), and CuSO_4_. After 48 hours, the treatments were removed from the fibroblasts and replaced with fibroblast growth media with charcoal-stripped serum containing 10 μM EdU and incubated for 2 hours at 37 °C and 5% CO_2_. The fibroblasts were then fixed for 10 minutes at room temperature (RT) by adding 500 μl of 4% paraformaldehyde (PFA) (Sigma-Aldrich, St. Louis, MO, USA, P6148-500G) directly to the media for a final concentration of 2% PFA. The PFA was removed, and the fibroblasts were washed 3 x 5–10 minutes with DPBS (Life Technologies/Gibco, Waltham, MA, USA, 14190-144). The fibroblasts were then permeabilized at RT for 5 minutes using 0.5% Triton X (Bio-Rad Laboratories, Hercules, CA, 1610407) in DPBS to facilitate EdU detection. Fibroblasts were then washed once with DPBS for 5–10 minutes before the detection step.

For each ml of EdU detection solution, the following reagents were added in order: 2 μl sulfo-cyanine 5 azide (2 mM, 500X), 658 μl autoclaved Millipore water, 100 μl Tris-buffered saline (1 M, pH 7.6), 40 μl CuSO_4_ (100 mM), and 200 μl sodium ascorbate (500 mM, 5X, 99 mg/ml). The sodium ascorbate was made fresh by dissolving 99 mg of L-ascorbic acid sodium salt powder (Thermo Scientific, Waltham, MA, USA, 352680050) in 1 ml of autoclaved Millipore water. 100 μl of EdU detection solution were added to each well, and the chamber slides were incubated in the dark at RT for 30 minutes. The fibroblasts were then washed 3 x 5–10 minutes with DPBS, the chambers were removed from the slides, and the slides were cover-slipped with DAPI (1 μg/ml) (Sigma-Aldrich, St. Louis, MO, USA, D9564-10MG) in Vectashield (Vector Laboratories, Inc., Newark, CA, USA, H-1000) as the mounting media. The slides were imaged using a Nikon NiE microscope and the number of EdU-positive fibroblasts were quantified using Nikon NIS-Elements Basic Research software.

### Scratch migration assay

Scratch migrations assays were performed using passage 2 fibroblasts isolated from quadriceps (adult) or hindlimb (neonatal) skeletal muscle from adult (8-week-old) C57 wild-type mice (n= 4; 2 M, 2 F), adult (8-week-old) *mdx* mice (n = 4; 2 M, 2 F), neonatal C57 wild-type mice (n = 4; 2 M, 2 F), and neonatal *mdx* mice (n = 4; 2 M, 2 F). 48 hours before treatment, fibroblasts were trypsinized and counted, and either 100,000 (neonatal) or 200,000 (adult) fibroblasts were plated into each well of a 12-well plate. Twenty-four hours before treatment, the fibroblasts were incubated at 37 °C and 5% CO_2_ in fibroblast growth media containing 10% charcoal-stripped fetal bovine serum (FBS). Fibroblasts were pre-treated for 24 hours with either aldosterone (1 μM), vehicle (DMSO), or 10 ng/ml TGF-β1 as a positive control. One well of the 12-well plate did not have cells, serving as a no-cell control to account for possible contamination of the media. One well containing cells was used as a no-treatment, no-scratch control.

After 24 hours of pre-treatment when the fibroblasts were 80–90 percent confluent, the fibroblasts were starved for 2 hours at 37 °C and 5% CO_2_ using serum-free media containing DMEM and 1% penicillin-streptomycin. After 2 hours, the fibroblasts were scratched using a 200 μl pipette tip to create a wound. The fibroblasts were then washed with warm sterile DPBS to remove dead cells and debris, and fresh charcoal-stripped media and treatments were added. The scratch wounds were then imaged using an Agilent Biotek Cytation 1 Multimode Plate Reader (Agilent Technologies, Santa Clara, CA, USA, CYT1VSN) every 12 hours for a period of 72 (neonatal) or 96 (adult) hours, at which time the wounds were mostly filled by migrating fibroblasts.

Wound area was quantified at each timepoint using ImageJ/Fiji^®^. The borders of the wounds were outlined using the tracing tool in ImageJ, and the wound area was measured using the updated wound healing size tool plug-in (Suarez-Arnedo et al., 2020). Average healing speed (μm^2^/h) was measured as the change in wound area at each timepoint relative to the 0h timepoint. Percent wound closure was measured as the percentage of the original wound area remaining at each timepoint relative to the wound area at the 0h timepoint, which was designated as 100% for each sample. Relative wound area was measured as the ratio of wound area at each timepoint relative to wound area at 0h.

### Statistical analysis

For the bulk RNA sequencing, Novogene used DESeq2 with p_adj_ ≤ 0.05 and |log_2_ fold change| ≥ 1 to determine significantly different gene expression. For the western blots, ratio paired t-tests were used to determine differences in protein expression, with *P* ≤ 0.05 considered significant. These t-tests were used to compare differences in protein level between paired fibroblast samples that were isolated from the same mice and treated with either aldosterone or vehicle. For the EdU proliferation assay, unpaired Student’s t-tests were used to determine differences in proliferation for vehicle- and aldosterone-treated fibroblasts within each genotype/age group (C57 neonatal, C57 adult, *mdx* neonatal, *mdx* adult), and unpaired Student’s t-tests were used to determine differences in proliferation between neonatal and adult samples grouped within each genotype, with *P* ≤ 0.05 considered significant. For each scratch migration assay, two-way repeated-measures ANOVA was used with Tukey’s multiple comparisons test to compare differences in average healing speed, percent wound closure, and relative wound area, with *P* ≤ 0.05 considered significant. Statistical analyses for western blots, proliferation and migration assays were performed using GraphPad Prism (GraphPad, San Diego, CA, USA).

## Results

### Direct aldosterone treatment induces gene expression changes in fibroblasts isolated from neonatal wild-type skeletal muscles

**Figure 1:**
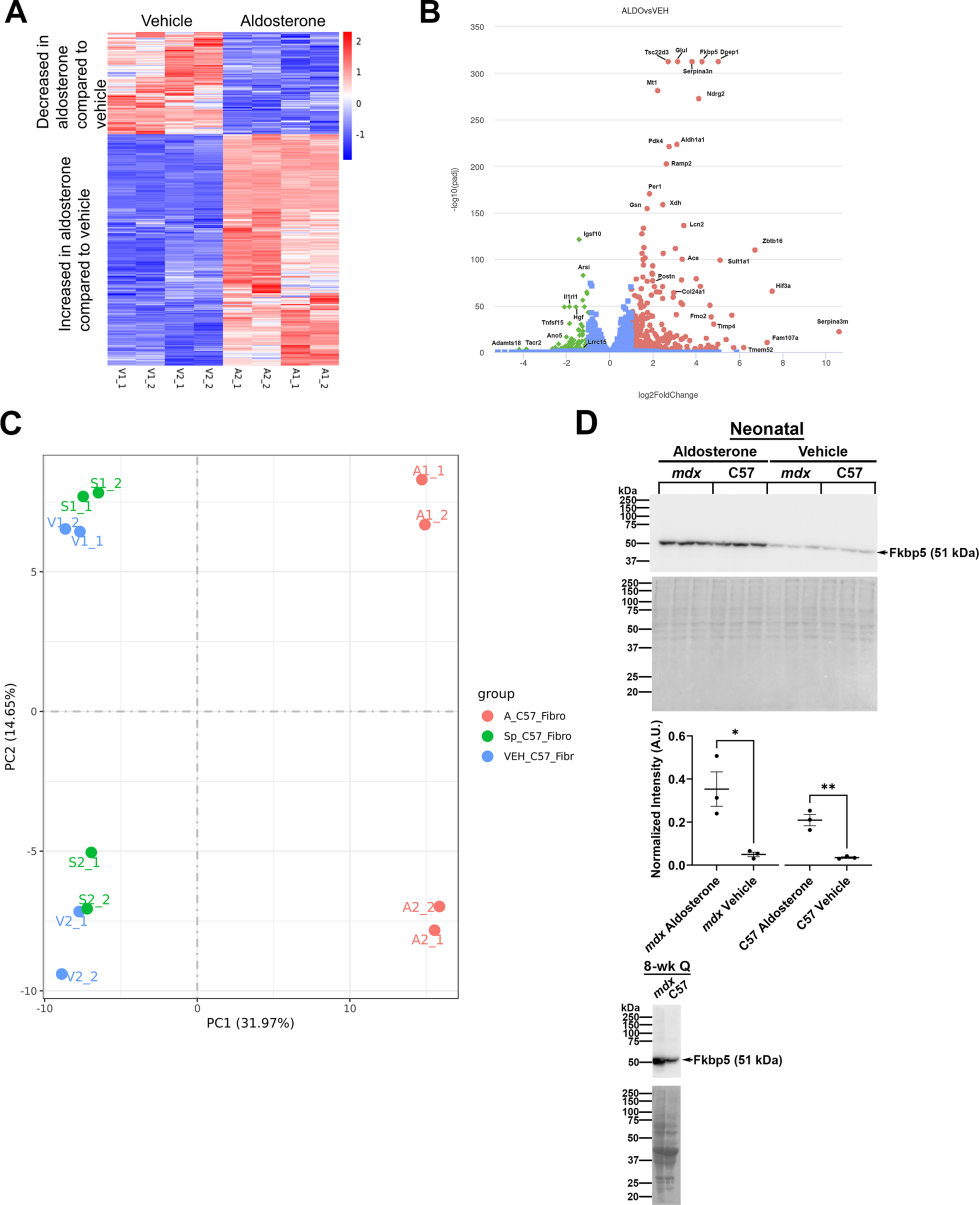
Bulk RNA-sequencing shows direct effects of aldosterone on gene expression in wild-type neonatal skeletal muscle fibroblasts. **(A)** Heatmap showing the number of genes increased and decreased with aldosterone treatment of fibroblasts isolated from hindlimb skeletal muscles of C57BL/10 neonatal mice. 341 genes were significantly increased by aldosterone treatment while 151 genes were decreased (p_adj_ ≤ 0.05 and |log_2_ fold change| ≥ 1). Genes were clustered based on normalized Z-scores (|Z-scores| ≤ 2), which were calculated for FPKM (Fragments Per Kilobase of transcript per Million mapped reads) values. **(B)** Volcano plot with significantly up- and downregulated genes color-coded in red (increased by aldosterone) and green (decreased by aldosterone) showing the identity of genes with the largest fold-changes and most significant p_adj_ value. **(C)** Principal component analysis (PCA) plot showing clustering of aldosterone-, spironolactone-, and vehicle-treated fibroblast RNA expression in bulk RNA-sequencing. **(D)** Western blot of aldosterone- and vehicle-treated hindlimb skeletal muscle fibroblasts from neonatal *mdx* and C57 wildtype mice, showing protein levels of Fkbp5 (top panel). Total protein via Ponceau S staining was used for normalization for Fkbp5. Ratio paired t-tests after quantification of band intensities with normalization showed significant differences between Fkbp5 protein levels between *mdx* aldosterone- and vehicle-treated fibroblasts and between C57 aldosterone- and vehicle-treated fibroblasts (middle panel). Fkbp5 also appears at higher levels in western blots of quadriceps muscle protein homogenates from 8-week-old *mdx* dystrophic muscles compared to wild-type C57 muscles (bottom panel). *P ≤ 0.05. **P ≤ 0.01.

Since the effects of direct aldosterone treatment on skeletal muscle fibroblast function and behavior are not known, we performed bulk RNA sequencing of naïve, neonatal fibroblasts isolated from C57BL/10 wild-type (C57) hindlimb skeletal muscles. Fibroblasts were treated at passage 2 with either the MR agonist aldosterone, the MR antagonist spironolactone, or vehicle for 48 hours and then RNA was isolated for differential gene expression analysis via deep sequencing. Aldosterone compared to vehicle treatment led to increased expression of 341 genes and reduced expression of 151 genes (p_adj_ ≤ 0.05, |log_2_ fold change| ≥ 1), demonstrating that fibroblast MR signaling directly affects skeletal muscle fibroblast gene expression (**Figures 1A, B**). Some of the greatest-fold-change physiologically relevant genes increased by aldosterone treatment include: *FK506-binding protein 51* (*Fkbp5*); *gelsolin* (*Gsn*); *serine peptidase inhibitor, clade A, member 3M* (*Serpina3m*); *serine peptidase inhibitor, clade A, member 3N* (*Serpina3n*); *period circadian clock 1* (*Per1*); *tissue inhibitor of metalloproteinase 4* (*Timp4*); and *hypoxia inducible factor 3 subunit alpha* (*Hif3a*) (**Supplementary Table 1**). These genes were considered physiologically relevant because of their previously documented roles in skeletal muscle physiology and pathophysiology. As expected, given that the spironolactone- and vehicle-treated samples clustered together in the principal component analysis (PCA) plot (**Figure 1C**), spironolactone treatment did not result in any gene expression changes compared to vehicle. Since charcoal-stripping of the serum used to make the culture media removes all endogenous steroids including aldosterone and corticosterone/cortisol that could activate MR, spironolactone treatment was utilized as a control for endogenous steroid removal.

Gene set enrichment analysis (GSEA) of all changed genes in aldosterone- versus vehicle-treated fibroblasts revealed that 207 gene sets were activated by aldosterone treatment and 135 gene sets were suppressed. After consolidation of semantically similar categories using GOSemSim and hierarchical clustering threshold of 0.7, 74 gene sets were activated by aldosterone and 41 were suppressed. The most significantly activated pathways with aldosterone were related to metabolism- and extracellular matrix-related processes. (**Supplementary Figure 2**). Aldosterone suppressed gene sets involved in proliferation-, migration-, and inflammation-related processes (**Supplementary Figure 2**). We previously identified that expression of 17 genes belonging to a set of genes that characterize myofibroblasts across several organs were upregulated in skeletal muscles from *mdx* mice containing a myeloid MR knockout compared with a myofiber MR knockout (Gomatam et al., 2024; Buechler et al., 2021). Of these, direct aldosterone activation of MR only increased *periostin* (*Postn*) and *cartilage intermediate layer protein* (*Cilp*), suggesting that they possibly drive early development of fibrosis in skeletal muscle (Trundle et al., 2024). Interestingly, *leucine rich repeat containing 15* (*Lrrc15*), which was the marker of the cluster of myofibroblasts, was actually decreased by aldosterone treatment of C57 wild-type neonatal fibroblasts (Buechler et al., 2021).

To validate our RNA sequencing results and confirm increased gene expression was translated into increased protein expression, we performed a western blot for a representative important target. *Fkbp5* was increased by 19-fold by aldosterone compared to vehicle treatment of neonatal wild-type fibroblasts (**Supplementary Table 1**). Fkbp5 contributes to the regulation of the aldosterone-driven stress response by conferring stability of MR in the cytoplasm to prevent its translocation to the nucleus (Petrovich et al., 2014; Ueda et al., 2014; Zannas et al., 2016). Fkbp5 is a known direct transcriptional target of MR in other cell types, supporting it is an important part of the self-regulation of the MR stress response (Gao et al., 2023; Hartmann et al., 2021; Petrovich et al., 2014). It is also known to negatively modulate glucocorticoid receptor sensitivity and its expression is reduced in late stage human DMD skeletal muscle (Gao et al., 2023).

We assessed Fkbp5 levels between aldosterone- and vehicle-treated C57 wild-type and *mdx* neonatal skeletal muscle fibroblasts to determine whether these cells exhibit differential responses to aldosterone treatment. Fkbp5 levels were significantly higher in aldosterone-treated *mdx* fibroblasts than in vehicle-treated *mdx* fibroblasts (0.35 ± 0.08 A.U. vs. 0.05 ± 0.01 A.U., *P* ≤ 0.05) and in aldosterone-treated C57 compared to vehicle-treated C57 fibroblasts (0.21 ± 0.03 A.U. vs. 0.04 ± 0.004 A.U., *P* ≤ 0.01) (**Figures 1D**, top and middle panels). We validated the Fkbp5 antibody using protein from adult (8-wk) C57 and *mdx* quadriceps muscles and observed that Fkbp5 protein levels were increased in the *mdx* quadriceps compared to C57 supporting its disease relevance (**Figure 1D**, bottom panel).

### Aldosterone represses proliferation in *mdx* adult skeletal muscle fibroblasts

**Figure 2:**
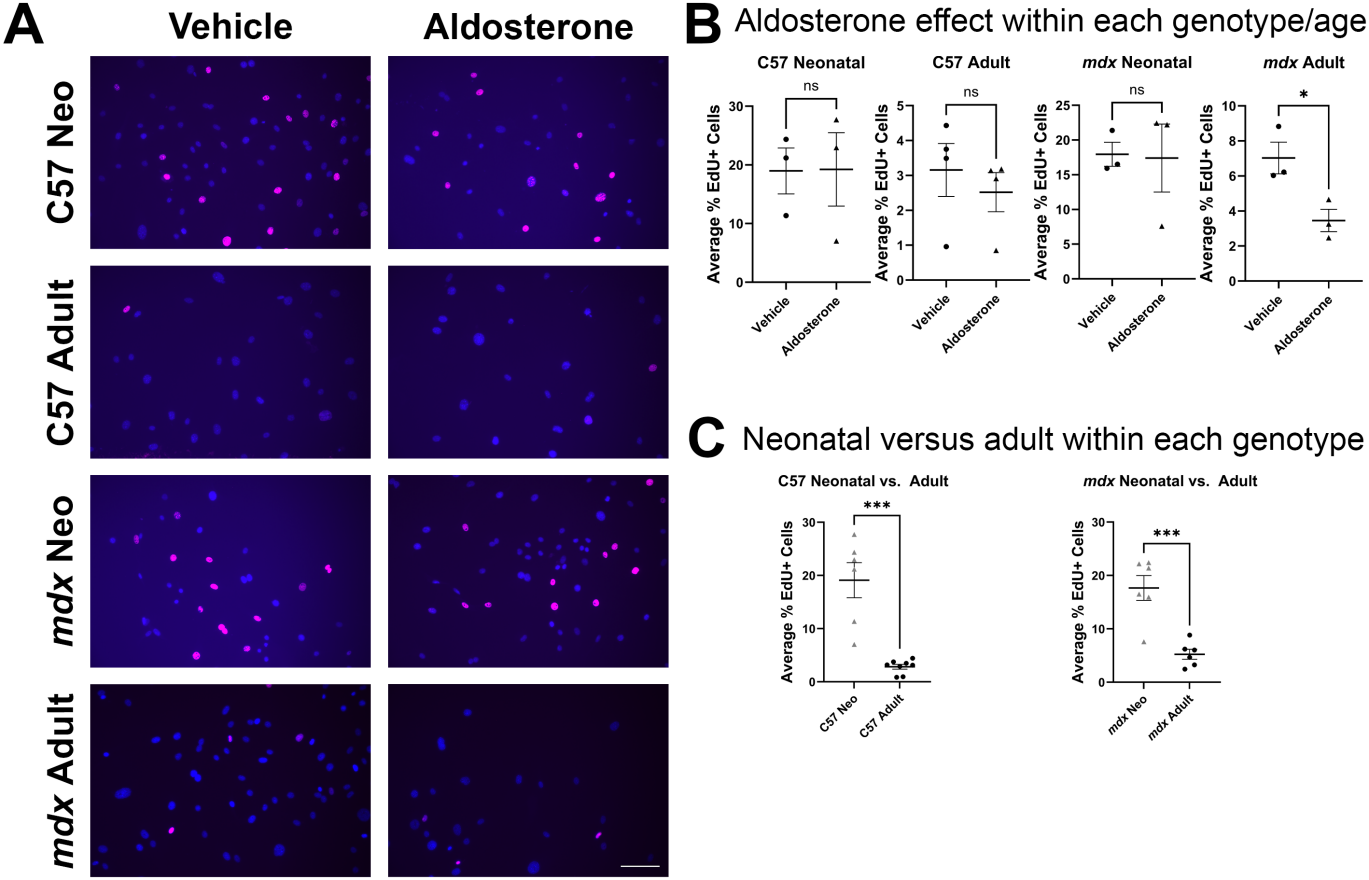
Neonatal skeletal muscle fibroblasts retain a higher proliferation rate in culture compared with adult skeletal muscle fibroblasts and aldosterone represses proliferation only in adult *mdx* fibroblasts. **(A, B)** Representative immunofluorescence images and quantification of an EdU proliferation assay comparing fibroblasts isolated from adult (8-week-old) quadriceps and neonatal hindlimb muscle from *mdx* and C57 mice treated with vehicle or aldosterone. **(A)** Representative images of DAPI- and EdU-positive nuclei of vehicle- and aldosterone-treated fibroblasts from neonatal and adult C57 and *mdx* samples. **(B)** Unpaired Student’s t-tests showed significant differences in proliferation with aldosterone treatment only for adult *mdx* fibroblasts. Aldosterone did not significantly affect fibroblast proliferation in neonatal and adult C57 fibroblasts or neonatal *mdx* fibroblasts. The graphs display results of unpaired Student’s t-tests comparing means between vehicle- and aldosterone-treated fibroblasts for each genotype/age group. Treatment of fibroblasts with the mineralocorticoid receptor antagonist spironolactone showed no quantitative differences from vehicle (not shown) and served as a control for the removal of endogenous steroids from charcoal-stripped serum in the culture media. **(C)** Unpaired student’s t-tests demonstrate neonatal fibroblasts for each genotype (C57 left, *mdx* right) are more proliferative compared to adult fibroblasts from the same genotype. Aldosterone- and vehicle-treated fibroblasts were grouped for each genotype for this comparison. *P ≤ 0.05. *** *P* ≤ 0.001. Bar = 100 µm.

Since direct stimulation of fibroblast MR with aldosterone changed gene expression in neonatal wild-type fibroblasts, we then tested whether aldosterone stimulation led to differential functional responses in fibroblasts involved in muscle growth, maintenance or disease. We cultured fibroblasts from both neonatal and adult C57 wild-type and dystrophic *mdx* skeletal muscles and measured fibroblast proliferation activity. We used EdU to mark proliferating cells after a 48-hour treatment of cultured muscle fibroblasts with aldosterone or vehicle and compared the effect of aldosterone treatment within each cell population (**Figure 2A**). There were no significant differences in proliferation between vehicle- and aldosterone-treated C57 neonatal fibroblasts (19.0 ± 3.91% EdU^+^ cells vs. 19.2 ± 6.25% EdU^+^ cells, *P* = 0.97), between vehicle- and aldosterone-treated C57 adult fibroblasts (3.16 ± 0.76% EdU^+^ cells vs. 2.52 ± 0.56% EdU^+^ cells, *P* = 0.52) nor between vehicle- and aldosterone-treated *mdx* neonatal fibroblasts (17.9 ± 1.73% EdU^+^ cells vs. 17.4 ± 4.90% EdU^+^ cells, *P* = 0.92) (**Figure 2B**). However, vehicle-treated *mdx* adult fibroblasts had a significantly higher rate of proliferation than aldosterone-treated *mdx* adult fibroblasts (7.03 ± 0.90% EdU^+^ cells vs. 3.46 ± 0.64% EdU^+^ cells, *P* = 0.032) (**Figure 2B**). We observed that neonatal fibroblasts appeared more proliferative than adult fibroblasts regardless of genotype or treatment, and since we performed the treatment and EdU assay on all the fibroblast samples at the same time, we compared the proliferation of C57 neonatal fibroblasts with C57 adult fibroblasts and *mdx* neonatal fibroblasts with *mdx* adult fibroblasts. Neonatal fibroblasts showed more proliferation compared to adult fibroblasts for both wild-type and dystrophic *mdx* mice (C57 neonatal: 19.1 ± 3.30% EdU^+^ cells vs. C57 adult: 2.84 ± 0.45% EdU^+^ cells, *P* ≤ 0.001; *mdx* neonatal: 17.7 ± 2.33% EdU^+^ cells vs. *mdx* adult: 5.24 ± 0.94% EdU^+^ cells, *P* ≤ 0.001). This data demonstrates that skeletal muscle fibroblasts in culture after 2 passages retain *in vivo* properties such as a higher proliferative rate in neonatal muscles (**Figure 2C**). Fibroblast samples from all four groups treated with TGF-β as a positive control before EdU incorporation were not quantified, but images show similar percentages of EdU as aldosterone- and vehicle-treated fibroblasts (**Supplementary Figure 3**).

### Aldosterone may also repress migration in *mdx* adult skeletal muscle fibroblasts

**Figure 3:**
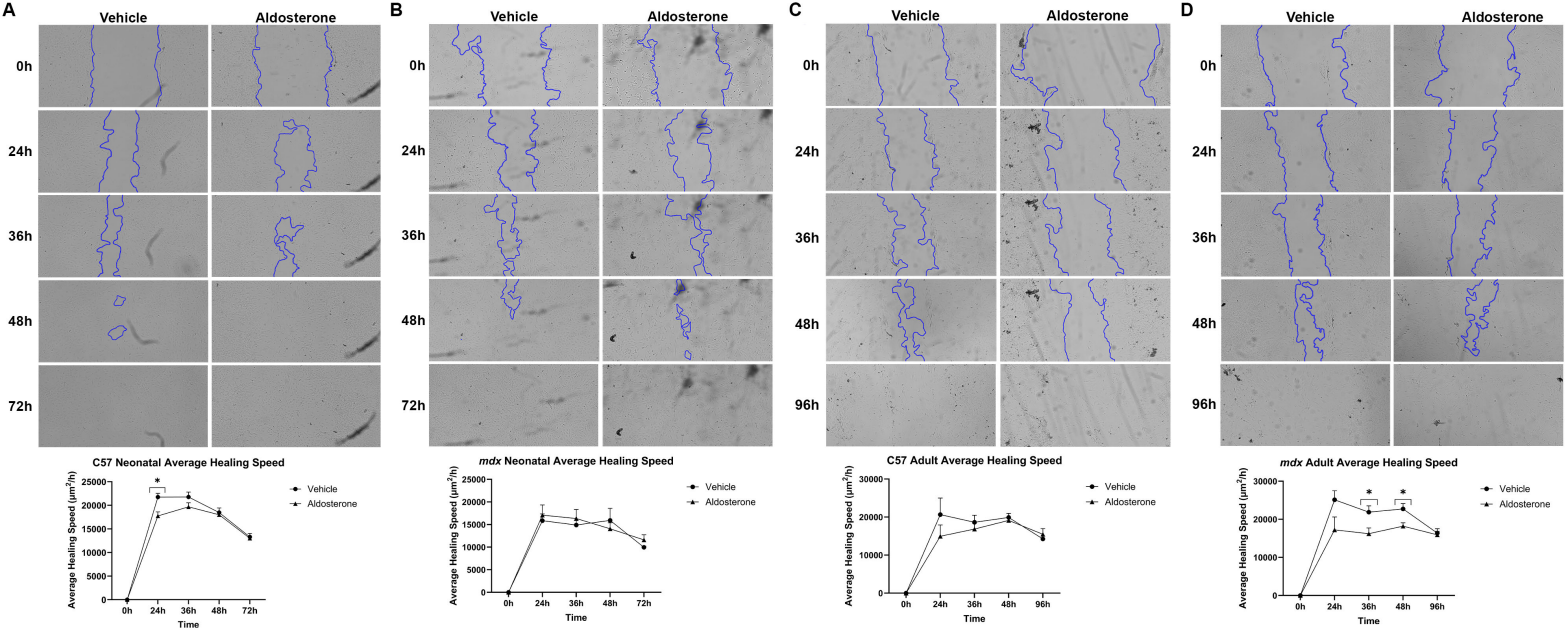
Aldosterone reduces wound healing speed of *mdx* adult skeletal muscle fibroblasts in an *in vitro* motility assay. Representative images showing the change in scratch wound area over 72–96 hours for vehicle- and aldosterone-treated fibroblasts isolated from adult (8-week-old) quadriceps and neonatal hindlimb muscle from *mdx* and C57 mice. **(A)** Representative images of scratch wound area over 72 hours for fibroblasts from C57 neonatal hindlimb skeletal muscle (top panel). No significant differences were observed in the average healing speed (µm^2^/h) between aldosterone- and vehicle-treated fibroblasts (bottom panel). **(B)** Representative images of scratch wound area over 72 hours for fibroblasts from *mdx* neonatal hindlimb skeletal muscle (top panel). No significant differences were observed in the average healing speed (µm^2^/h) between aldosterone- and vehicle-treated fibroblasts (bottom panel). **(C)** Representative images of scratch wound area over 96 hours for fibroblasts from C57 adult quadriceps (top panel). No significant differences were observed in the average healing speed (µm^2^/h) between aldosterone- and vehicle-treated fibroblasts (bottom panel). **(D)** Representative images of scratch wound area over 96 hours for fibroblasts from *mdx* adult quadriceps (top panel). Aldosterone-treated fibroblasts exhibited significantly slower average healing speed (µm^2^/h) than vehicle-treated fibroblasts at the 36- and 48-hour timepoints (bottom panel). TGF-β treatment was used as a positive control in all 4 groups (see **Supplementary Figure 3**).

Fibroblasts’ ability to migrate to an injury site is crucial for their role in stabilizing the wound area and generating a scaffold to allow for satellite cell-mediated regeneration of muscle fibers. To test the effects of direct aldosterone treatment on fibroblast migration, we performed *in vitro* scratch assays using C57 wild-type and *mdx* dystrophic skeletal muscle fibroblasts isolated from adult and neonatal mice. Fibroblasts from each group were treated with either aldosterone or vehicle and then a scratch was used to clear an area between cells to allow the fibroblasts to migrate into the area and close the gap. The wound area was imaged every 12 hours for 72 hours (neonatal fibroblasts) or 96 hours (adult fibroblasts) to monitor the closure of the gap by the migrating fibroblasts (**Figure 3**). We assessed several different measurements of wound healing and fibroblast migration, including percent wound closure, the relative change in wound area, and the change in healing speed (μm^2^/h). The healing speed was significantly slower in aldosterone-treated C57 neonatal fibroblasts compared to vehicle-treated C57 neonatal fibroblasts at the 24-hour timepoint (17,748 ± 1736 μm^2^/h vs. 21,745 ± 1603 μm^2^/h; *P* ≤ 0.05) but was not significantly different at any other timepoint (**Figure 3A**). The healing speed was not significantly different between aldosterone- and vehicle-treated *mdx* neonatal fibroblasts (**Figure 3B**), nor between aldosterone- and vehicle-treated C57 adult fibroblasts (**Figure 3C**). In contrast, the healing speed was significantly slower in aldosterone-treated *mdx* adult fibroblasts at the 36- and 48-hour timepoints compared to vehicle-treated *mdx* adult fibroblasts (**36** h: 16,239 ± 1489 μm^2^/h vs. 21,888 ± 1655 μm^2^/h; 48 h: 18,225 ± 853 μm^2^/h vs. 22,736 ± 1407 μm^2^/h; *P* ≤ 0.05) (**Figure 3D**). The percent wound closure and relative wound area were not significantly different between aldosterone and vehicle-treatment in any of the four fibroblast cultures. It is important to note that proliferation was not inhibited in these assays, meaning that fibroblast proliferation may have contributed to the closure of the scratch wound along with migration. Nevertheless, these data suggest that aldosterone may have a repressive effect on migration as well as proliferation in adult *mdx* skeletal muscle fibroblasts. Fibroblast samples were treated with TGF-β prior to the scratch as positive controls, and the wounds closed completely over 72–96 hours for TGF-β-treated samples from all four groups (**Supplementary Figure 3**).

### Changes in protein levels of proliferation-related genes provide potential mechanistic insight into MR signaling in skeletal muscle fibroblasts

To further explore the mechanisms by which aldosterone affects fibroblast function, we investigated protein levels of additional key genes from our bulk RNA sequencing data that are directly involved in proliferation. Through our gene set enrichment analysis (GSEA), we found that *cyclin-dependent kinase inhibitor 1c* (*Cdkn1c*) and *FBJ osteosarcoma oncogene* (*Fos*) were present in activated gene sets associated with decreased proliferation. *Cdkn1c* encodes the protein p57 Kip2 (p57), which is a negative regulator of cell proliferation in various cell types (Sherr and Roberts, 1999; Besson et al., 2008). p57 has also been shown to have noncanonical roles that may also affect fibroblast function, such as context-dependent stimulation or inhibition of apoptosis (Rossi and Antonangeli, 2015; Creff and Besson, 2020). c-Fos, the protein product of the *Fos* gene, is a component of the Activator Protein-1 (AP-1) transcription factor, which regulates many cellular processes including the cell cycle (Shaulian and Karin, 2001). c-Fos typically enhances cell proliferation but can act in the opposite manner under some conditions (Oliveira-Ferrer et al., 2014; Mikula et al., 2003; Mahner et al., 2008). In our bulk RNA sequencing of aldosterone- versus vehicle-treated neonatal wild-type fibroblasts, Cdkn1c was increased 5.8-fold and Fos was increased 2.1-fold (**Supplementary Table 1**). We performed western blot analyses to compare protein levels of p57 and c-Fos between aldosterone and vehicle-treated fibroblasts isolated from C57 and *mdx* neonatal hindlimb and adult quadriceps skeletal muscle.

**Figure 4:**
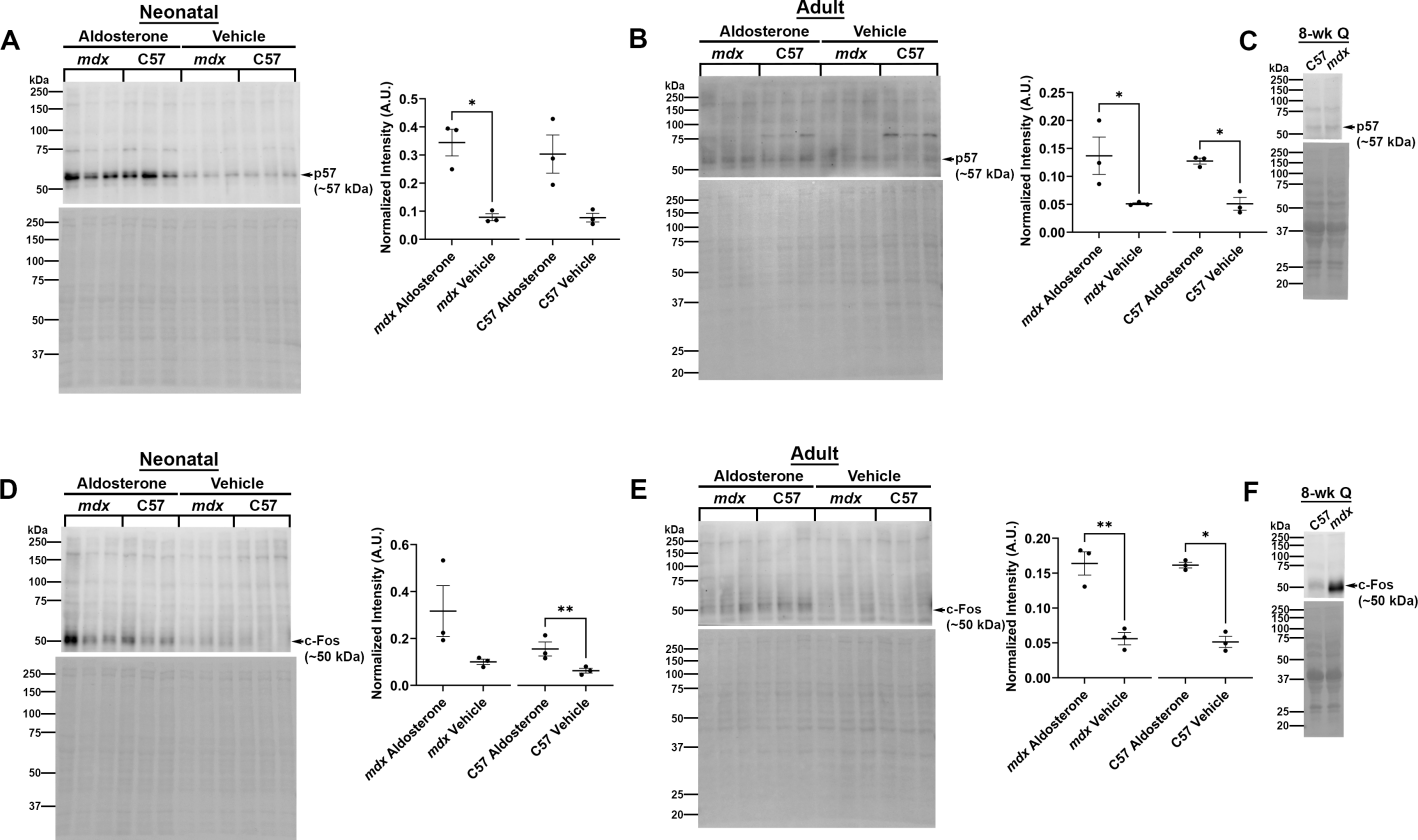
p57 and c-Fos protein levels are increased by aldosterone treatment of neonatal and adult skeletal muscle fibroblasts. Western blots for p57 **(A–C)** and c-Fos **(D–F)** of aldosterone- and vehicle-treated hindlimb skeletal muscle fibroblasts from neonatal *mdx* and C57 wild-type mice [**(A, D)**, left panels], and of aldosterone- and vehicle-treated quadriceps skeletal muscle fibroblasts from adult *mdx* and C57 wild-type mice [**(B, E)**, left panels]. Total protein via Ponceau S staining was used for normalization for both p57 and c-Fos. Quantification of band intensities after normalization is shown for all western blots [**(A, B, D, E)**, right panels]. Ratio paired t-tests showed significant differences for: p57 protein levels between neonatal *mdx* aldosterone- and vehicle-treated fibroblasts, between adult *mdx* aldosterone- and vehicle-treated fibroblasts, and between adult C57 aldosterone- and C57 vehicle-treated fibroblasts **(A, B)**. c-Fos protein levels were significantly different between neonatal C57 aldosterone- and vehicle-treated fibroblasts, between adult *mdx* aldosterone- and vehicle-treated fibroblasts, and between adult C57 aldosterone- and vehicle-treated fibroblasts **(D, E)**. p57 **(C)** and c-Fos **(F)** were also tested on quadriceps muscle protein homogenates from 8-week-old mice to compare relative levels between wild-type C57 and *mdx* dystrophic muscles. *P ≤ 0.05. **P ≤ 0.01.

p57 levels were significantly higher in aldosterone-treated neonatal *mdx* fibroblasts compared to vehicle-treated neonatal *mdx* fibroblasts (0.34 ± 0.05 A.U. vs. 0.08 ± 0.01 A.U., *P* ≤ 0.05) and trended higher in aldosterone-treated neonatal C57 fibroblasts compared to vehicle-treated neonatal C57 fibroblasts (0.31 ± 0.07 A.U. vs. 0.08 ± 0.02 A.U., *P* = 0.09) (**Figure 4A**). p57 levels were also significantly higher in aldosterone-treated adult *mdx* fibroblasts compared to vehicle-treated adult *mdx* fibroblasts (0.14 ± 0.03 A.U. vs. 0.05 ± 0.002 A.U., *P* ≤ 0.05) and in aldosterone-treated adult C57 fibroblasts compared to vehicle-treated adult C57 fibroblasts (0.13 ± 0.005 A.U. vs. 0.05 ± 0.01 A.U., *P* ≤ 0.05) (**Figure 4B**). p57 levels were not different between *mdx* and C57 quadriceps muscle homogenates (**Figure 4C**).

c-Fos protein levels were significantly higher in aldosterone-treated neonatal C57 fibroblasts compared to vehicle-treated neonatal C57 fibroblasts (0.15 ± 0.03 A.U. vs. 0.06 ± 0.01 A.U., *P* ≤ 0.01) (**Figure 4D**). c-Fos protein levels were also significantly higher in aldosterone-treated adult *mdx* fibroblasts compared to vehicle-treated adult *mdx* fibroblasts (0.16 ± 0.02 A.U. vs. 0.06 ± 0.009 A.U., *P* ≤ 0.01) and in aldosterone-treated adult C57 fibroblasts compared to vehicle-treated adult C57 fibroblasts (0.16 ± 0.004 A.U. vs. 0.05 ± 0.008 A.U., *P* ≤ 0.05) (**Figure 4E**). c-Fos levels also appeared higher in *mdx* compared to C57 whole quadriceps muscles (**Figure 4F**).

## Discussion

In this study, we show that direct aldosterone treatment of fibroblasts isolated from neonatal wild-type skeletal muscles changed the expression of almost 500 genes. We then expanded the study to investigate the short-term functional effects of aldosterone stimulation on proliferation and migration in neonatal and adult wild-type and dystrophic skeletal muscle fibroblasts and showed adult *mdx* fibroblasts were most susceptible to functional changes in both parameters.

Treating fibrotic diseases must involve finding a balance between maintaining fibroblasts’ wound healing ability while attenuating pathological fibrosis and inflammation. We have previously shown that MR antagonism can improve muscle function in dystrophic muscles in part by limiting fibrosis and inflammation, that indirect *in vivo* MR signaling from infiltrating immune cells and myofibers can affect fibrosis, and that indirect MR signaling from cultured myotubes differentially impacts fibroblasts from neonatal wild-type and *mdx* skeletal muscles (Chadwick et al., 2015; Howard et al., 2022b; Hauck et al., 2019b; Gomatam et al., 2024; Krishna et al., 2025). Therefore, understanding how fibroblast-specific MR signaling directly changes fibroblast function and the differences between fibroblasts isolated from muscle during growth, maintenance and disease is crucial for optimizing existing therapeutic approaches and potentially opening up new avenues for treatment of DMD and possibly other fibrotic muscle diseases.

*Postn* and *Cilp* were the only two myofibroblast genes upregulated by aldosterone treatment in wild-type neonatal skeletal muscle fibroblasts. Postn is a secreted extracellular matrix protein that plays a role in fibrosis development via increasing collagen deposition and a potential role in collagen cross-linking (Maruhashi et al., 2010; Trundle et al., 2024). Cilp1 promotes cardiac fibrosis after myocardial infarction and during cardiomyopathy through interaction with the TGF-β1 pathway (Zhang et al., 2021; Van Nieuwenhoven et al., 2017). *Cilp* gene expression is increased in human DMD skeletal muscle (Nieves-Rodriguez et al., 2023). Additionally, Cilp1 plays a significant role in skeletal muscle development (Ralliere et al., 2015). It is possible that these two genes may be responsible for an early fibrotic response since they are present at the developmental stage in wild-type mice, increased by the aldosterone stress response and have demonstrated profibrotic roles in adult tissues.

*Fkbp5* was highly increased at the protein level with aldosterone treatment in both neonatal wild-type and *mdx* fibroblasts. *Fkbp5* is a known MR transcriptional target that confers stability of MR in the cytoplasm to prevent its translocation to the nucleus and self-regulates the stress response (Petrovich et al., 2014; Ueda et al., 2014; Zannas et al., 2016). It is known to be downregulated in later stage human DMD skeletal muscle, allowing the potential for an unregulated MR and glucocorticoid receptor regulated stress response (Gao et al., 2023).

*Hypoxia inducible factor 3 subunit alpha* (*Hif3a*) was also upregulated with aldosterone treatment. In DMD, respiratory impairment can lead to hypoxia in skeletal muscle (Nguyen et al., 2021). Activation of hypoxia inducible factor 1α (HIF-1α) in dystrophic muscle is known to worsen pathology, but the role of hypoxia inducible factor 3 subunit alpha (HIF-3α)is less clear (Nguyen et al., 2021; Valle-Tenney et al., 2020). HIF-3α can negatively regulate HIF-1α, suggesting that it may reduce the negative effects of muscle ischemia (Duan, 2016).

Two members of the SerpinA clade of serine peptidase inhibitors (serpins) [mouse orthologs of human antichymotrypsin (*SERPINA3*)] (Horvath et al., 2005), *Serpina3m* and *Serpina3n*, were among the most highly upregulated genes expressed by the aldosterone-treated fibroblasts. SerpinA3 members are MR targets in the heart involved in tissue protection, play important roles in inflammatory diseases, and could be responsible for some side effects of long-term glucocorticoid treatment (Chadwick et al., 2015; Latouche et al., 2010; Lannan et al., 2012). We have previously shown that *Serpina3m* is downregulated in dystrophic mouse quadriceps after treatment with the MRA spironolactone (Chadwick et al., 2015). *Serpina3n* was upregulated in dystrophic skeletal muscle and after acute injury, and *Serpina3n* overexpression in dystrophic mouse models and after acute injury was shown to lead to stabilization of the sarcolemma, increased muscle regeneration, and decreased degeneration (Tjondrokoesoemo et al., 2016).

*Period circadian clock 1* (*Per1*) was also upregulated by aldosterone in fibroblasts. *Per1* expression is downregulated in both *mdx* mouse and human DMD skeletal muscle, part of a cascade of disruptive circadian rhythm changes that occur due to the loss of dystrophin, leading to impaired muscle regeneration and increased fibrosis, among other negative effects (Rossi et al., 2021; Betts et al., 2021). We have shown that both the MR antagonist spironolactone and the GR agonist prednisolone reduce *Per1* expression in myeloid cells isolated from quadriceps from treated *mdx* mice (Howard et al., 2022a). Though these genes likely play important roles in adult-stage DMD, their upregulation by aldosterone in wild-type neonatal skeletal muscle fibroblasts may not fully explain their roles in adult wild-type or dystrophic skeletal muscle fibroblasts, despite our observations that fibroblast function does seem to be affected by aldosterone in these fibroblasts.

Interestingly, neonatal and adult skeletal muscle fibroblasts exhibit different rates of proliferation regardless of genotype or treatment despite many days in culture, suggesting that they are resistant to phenotypic drift (Pan et al., 2009). Since functional changes in proliferation and migration were only observed in adult *mdx* fibroblasts, it is possible that aldosterone changes even more genes underlying these pathways in adult dystrophic fibroblasts compared to neonatal wild-type fibroblasts. The relative absence of proliferation and migration effects from aldosterone in wild-type neonatal fibroblasts may be due to a lack of sufficient sensitivity of these assays compared to transcriptomic signatures, particularly in cells that have such a high basal proliferation rate. Gene expression differences of MR-stimulated fibroblasts isolated from muscles during growth, maintenance and disease will need to be further evaluated in the future.

The repression of proliferation and migration by aldosterone contrasts with the effects of aldosterone on fibroblasts from other tissues. Sustained aldosterone expression in the heart increases proliferation and migration of cardiac fibroblasts, leading to increased fibrosis and hypertrophy (Somanna et al., 2015). Similarly, proliferation is increased in aldosterone-treated fibroblasts isolated from rat and mouse kidneys (Huang et al., 2012). It is possible that aldosterone-mediated repression of fibroblast proliferation and migration in adult dystrophic skeletal muscle could be driving the fibroblasts into a sedentary, ECM-producing myofibroblast state. Aldosterone appears to elicit these effects through different signaling pathways in different tissues, so understanding the pathways through which aldosterone acts in skeletal muscle could be important for designing therapies for skeletal muscle diseases (Somanna et al., 2015; Sakamuri et al., 2016; Huang et al., 2012; Stockand and Meszaros, 2003). A limitation of the scratch migration assays performed in the study was that proliferation was not inhibited. As such, it is difficult to determine how much fibroblast proliferation may have contributed to scratch closure compared to migration.

The differences in protein levels of known proliferation-associated genes *Cdkn1c* and *Fos* in response to aldosterone treatment could help explain the complex mechanisms by which aldosterone may act to mediate proliferation and other fibroblast functions. p57 and c-Fos were present at higher levels in aldosterone- versus vehicle-treated *mdx* adult skeletal muscle fibroblasts. p57 inhibits proliferation by inducing cell cycle arrest (Creff and Besson, 2020). Reduced levels of p57 are associated with Beckwith-Wiedemann Syndrome, which results in excessive growth (Weksberg et al., 2010). Although p57 can act as a proapoptotic protein, during stress p57 can instead promote survival (Chang et al., 2003). Since adult *mdx* fibroblasts are isolated from a stressed environment and aldosterone is a stress response hormone produced during muscle injury (Hauck et al., 2019a; Chadwick et al., 2016), p57-mediated inhibition of proliferation and promotion of survival during stress may contribute to reduced fibroblast proliferation in aldosterone-treated adult *mdx* fibroblasts and clearance-resistant myofibroblasts that contribute to aldosterone-exacerbated pathology in dystrophic muscles. In the heart, p57 is also known to act in a noncanonical manner during development. Instead of directly blocking the cell cycle through its induction of cell cycle arrest, p57 appears to regulate cell differentiation in the developing heart without necessarily causing cell cycle exit (Ponnusamy et al., 2017). This phenomenon may help explain why proliferation was not repressed by aldosterone in neonatal fibroblasts despite higher levels of p57 protein being present in aldosterone-treated fibroblasts.

As part of the Activating Protein-1 (AP-1) transcription factor complex, c-Fos regulates cell proliferation and differentiation and is known to be a target of aldosterone and MR-mediated signaling in multiple tissues (Okazaki and Sagata, 1995; Fiebeler et al., 2001; Latouche et al., 2010; Verrey et al., 2000). Aldosterone-induced activation of AP-1, which occurs through stimulation of the mitogen-activated protein kinase (MAPK)/extracellular signal-regulated kinase (ERK) pathway, increases *Col1a1* (*collagen, type I, alpha 1*) and leads to increased fibrosis in mice after cardiac injury (Fiebeler et al., 2001; Tharaux et al., 2000). Aldosterone also increases fibrosis following kidney injury through MR-mediated activation of c-Fos and AP-1 that increased fibroblast recruitment and activation to the myofibroblast state (Irita et al., 2008; Yoo et al., 2006). We have previously shown in quadriceps from dystrophic *mdx*;*utrn^+/−^* mice that *Fos* is reduced after 16 weeks of treatment with the MRA spironolactone (Chadwick et al., 2015). Similarly, in normal human skeletal muscle myotubes *FOS* is downregulated by spironolactone compared to aldosterone treatment (Chadwick et al., 2015, 2017). Though c-Fos is primarily known as an oncogene due to its requirement for proliferation, some studies show that aberrant c-Fos expression leads to inhibition of cell proliferation, suggesting that the temporal regulation and cellular environment of c-Fos is important for its complex role (Oliveira-Ferrer et al., 2014; Mikula et al., 2003; Mahner et al., 2008). ERK is also a known suppressor of p57 activity, so it is possible that MAPK/ERK-stimulated activation of AP-1 can also lead to suppression of p57, ultimately leading to increased proliferation (Worster et al., 2012).

It is worth noting that though all of our data is based on experiments performed using hindlimb muscle, the diaphragm develops the most severe fibrosis of all skeletal muscles in the *mdx* mouse (Stedman et al., 1991). Therefore, it would be beneficial to perform similar experiments on fibroblasts isolated from dystrophin-deficient diaphragms to determine whether these fibroblasts are different than those derived from other muscles.

Taken together, our data show that MR signaling may directly affect gene expression and function in skeletal muscle fibroblasts. It is possible that MR signaling triggered by the local production of aldosterone by macrophages (Chadwick et al., 2016) is repressing fibroblast proliferation and migration to allow them to remain in the injury site and secrete ECM proteins. Future combination treatment of fibroblasts with aldosterone and MR antagonists will be needed to absolutely confirm whether aldosterone and MR signaling are driving the observed transcriptional and functional changes in fibroblasts. Additional RNA sequencing of aldosterone- and vehicle-treated fibroblasts isolated from adult wild-type and *mdx* skeletal muscle, from neonatal *mdx* skeletal muscle, and from acute injury of wild-type skeletal muscle will further clarify the differences between skeletal muscle fibroblasts in the quiescent state in normal muscle, in wound healing, and in muscular dystrophy. This additional sequencing will also address an important limitation of our study that our gene expression data from wild-type neonatal fibroblasts may not fully explain our observations from the functional studies of adult fibroblasts and *mdx* neonatal fibroblasts. Additionally, MR-regulated crosstalk between immune cells and fibroblasts remains to be explored. Understanding the effects of MR signaling throughout the entire microenvironment is critical for clarifying the mechanism of action of existing MR antagonists and for discovering novel therapeutic approaches that can target chronic fibrosis but not compromise wound healing.

## Data Availability

The RNA sequencing data generated for this study can be found in the National Center for Biotechnology Information Gene Expression Omnibus (accession no. GSE297237).
