# Supplementary figures and images for: Direct aldosterone stimulation of skeletal muscle fibroblasts changes gene expression and differentially affects fibroblast functions from normal and diseased muscles
Source: Front Physiol. 2026 Apr 20;17:1760238. doi: 10.3389/fphys.2026.1760238 (PMC13135972; doi:10.3389/fphys.2026.1760238)

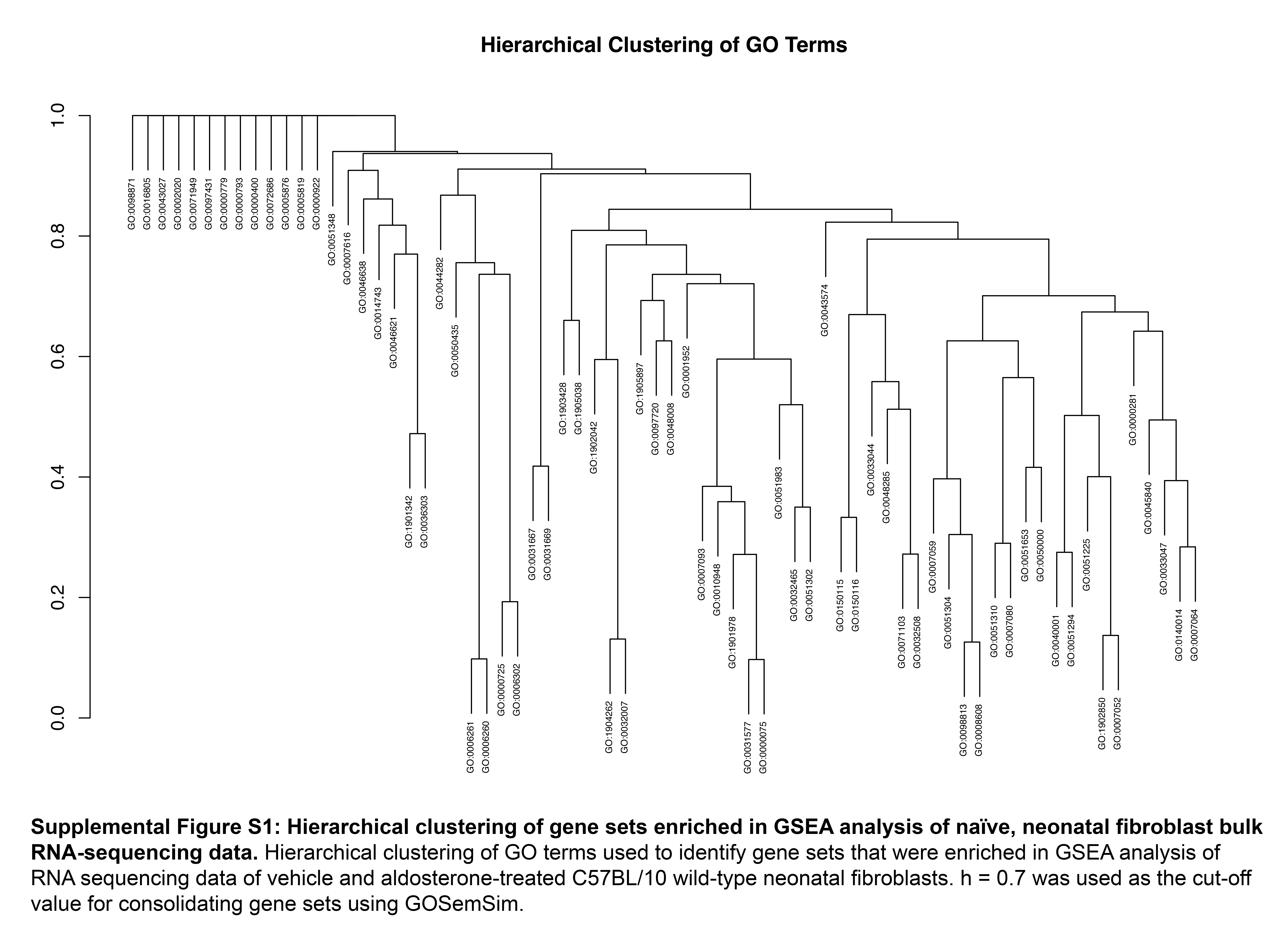

Supplement: Supplementary file 1 [file Image1.tif]

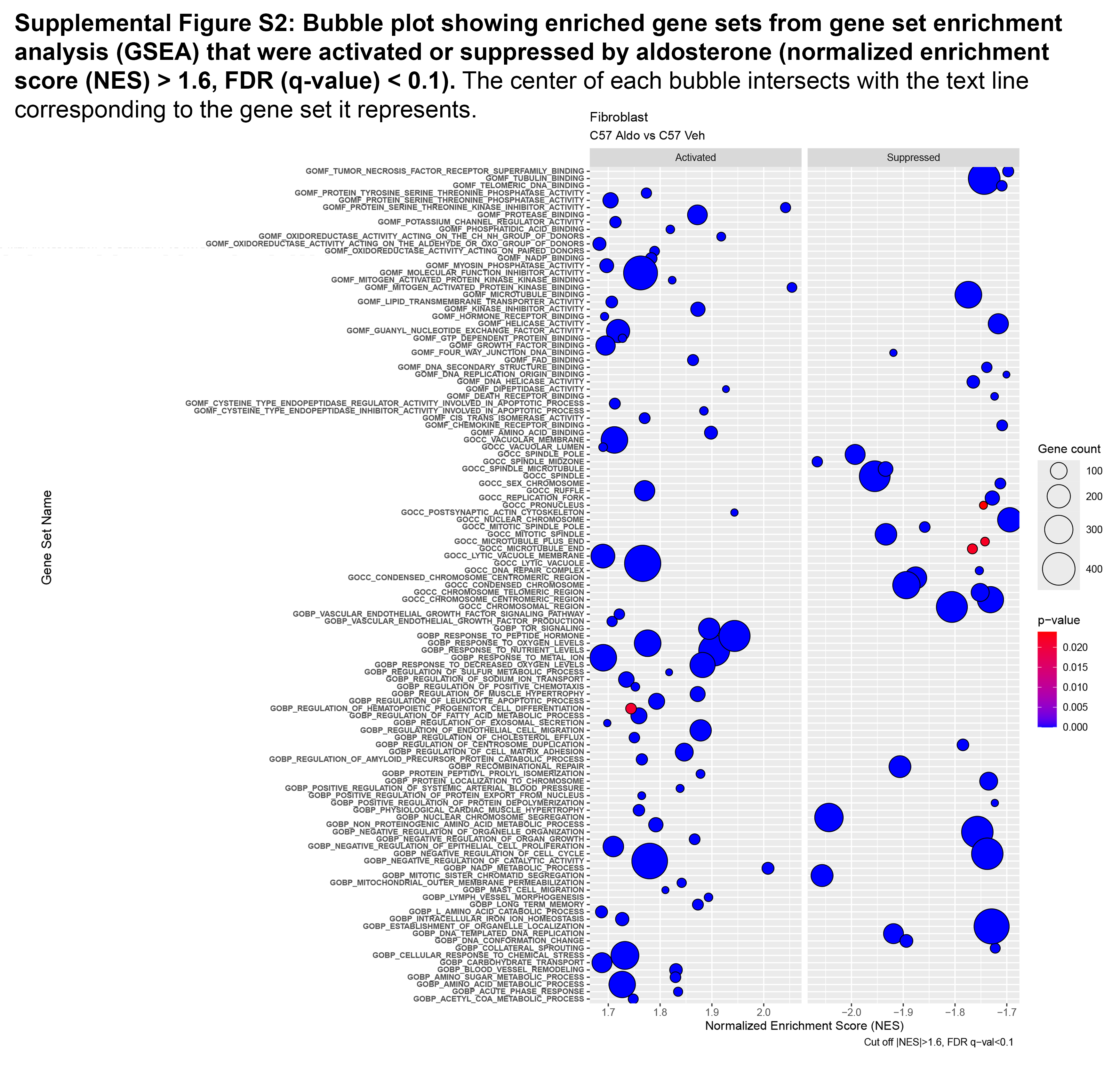

Supplement: Supplementary file 2 [file Image2.tif]

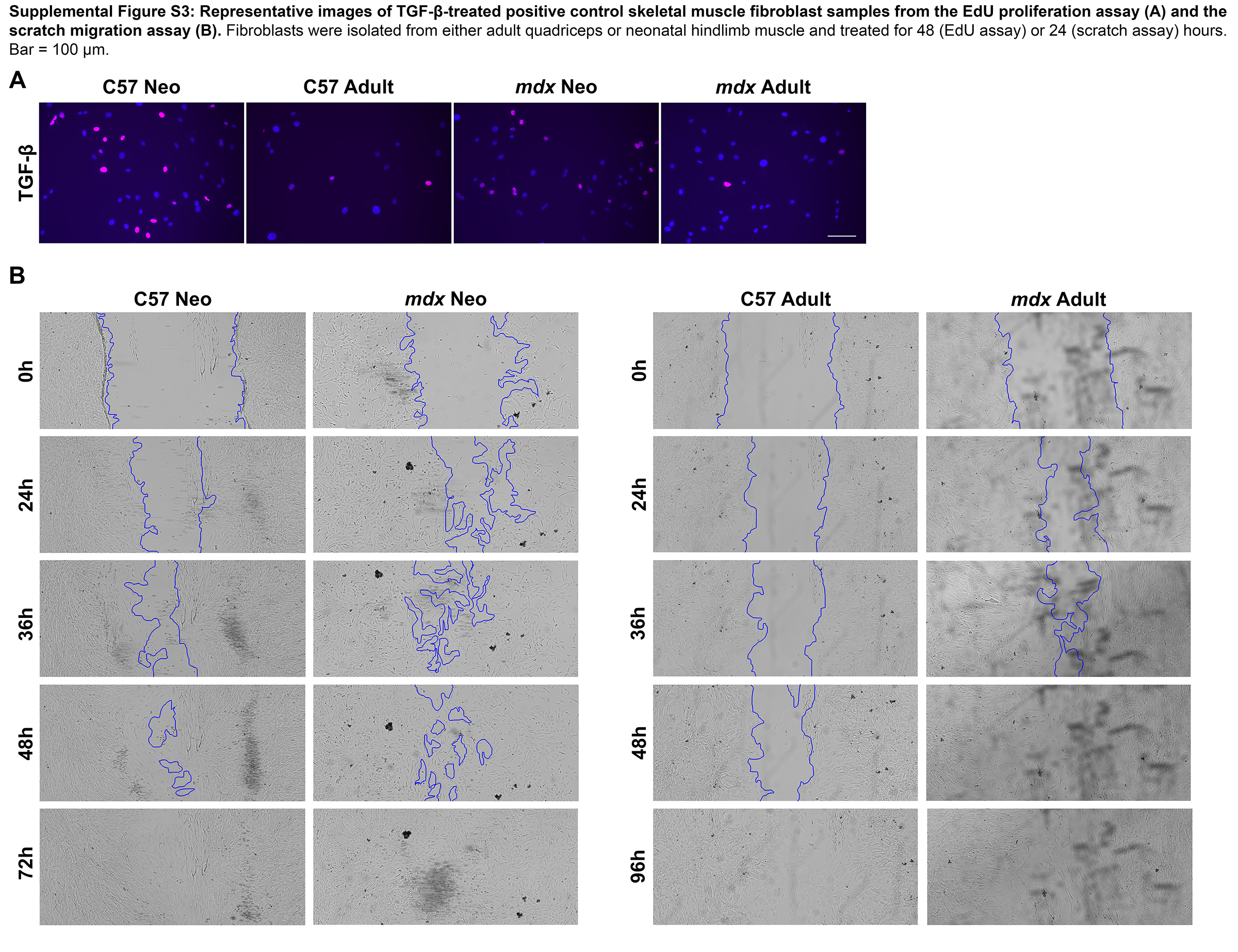

Supplement: Supplementary file 3 [file Image3.tif]

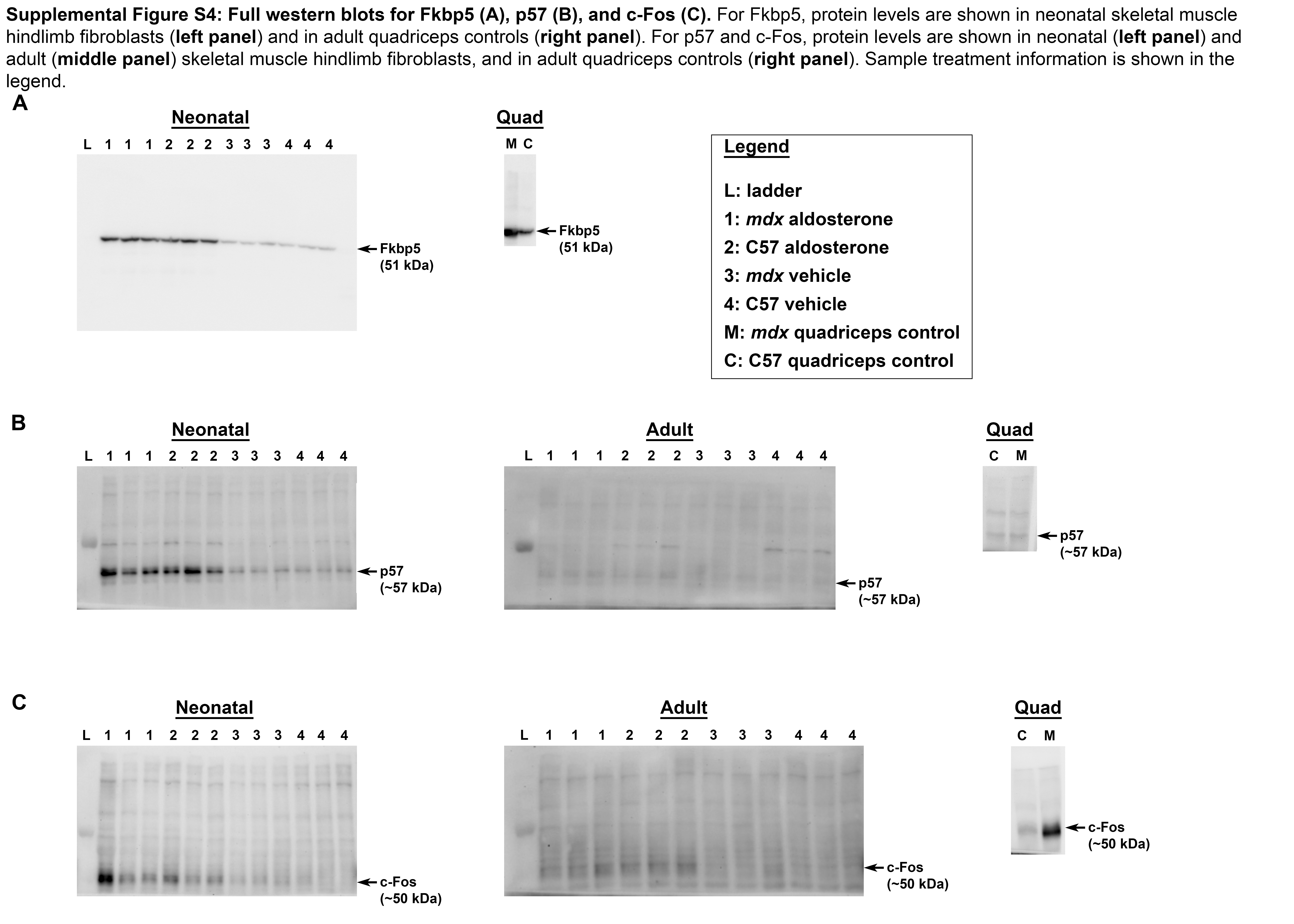

Supplement: Supplementary file 4 [file Image4.tif]
